# The multiple roles of histidine in protein interactions

**DOI:** 10.1186/1752-153X-7-44

**Published:** 2013-03-01

**Authors:** Si-Ming Liao, Qi-Shi Du, Jian-Zong Meng, Zong-Wen Pang, Ri-Bo Huang

**Affiliations:** 1 State Key Laboratory for Conservation and Utilization of Subtropical Agro-bioresources, Life Science and Biotechnology College, Guangxi University, Nanning, Guangxi, 530004, China; 2 State Key Laboratory of Non-food Biomass Energy and Enzyme Technology, National Engineering Research Center for Non-food Biorefinery, Guangxi Academy of Sciences, 98 Daling Road, Nanning, Guangxi, 530007, China; 3 Guangxi Mangrove Research Center, Beihai, Guangxi, 536000, China; 4 Gordon Life Science Institute, San Diego, California, 92130, USA

**Keywords:** Amino acids, Histidine, Protonation, Protein interaction, Protein structure

## Abstract

**Background:**

Among the 20 natural amino acids histidine is the most active and versatile member that plays the multiple roles in protein interactions, often the key residue in enzyme catalytic reactions. A theoretical and comprehensive study on the structural features and interaction properties of histidine is certainly helpful.

**Results:**

Four interaction types of histidine are quantitatively calculated, including: (1) Cation-π interactions, in which the histidine acts as the aromatic π-motif in neutral form (His), or plays the cation role in protonated form (His^+^); (2) π-π stacking interactions between histidine and other aromatic amino acids; (3) Hydrogen-π interactions between histidine and other aromatic amino acids; (4) Coordinate interactions between histidine and metallic cations. The energies of π-π stacking interactions and hydrogen-π interactions are calculated using CCSD/6-31+G(d,p). The energies of cation-π interactions and coordinate interactions are calculated using B3LYP/6-31+G(d,p) method and adjusted by empirical method for dispersion energy.

**Conclusions:**

The coordinate interactions between histidine and metallic cations are the strongest one acting in broad range, followed by the cation-π, hydrogen-π, and π-π stacking interactions. When the histidine is in neutral form, the cation-π interactions are attractive; when it is protonated (His^+^), the interactions turn to repulsive. The two protonation forms (and pK_a_ values) of histidine are reversibly switched by the attractive and repulsive cation-π interactions. In proteins the π-π stacking interaction between neutral histidine and aromatic amino acids (Phe, Tyr, Trp) are in the range from -3.0 to -4.0 kcal/mol, significantly larger than the van der Waals energies.

## Introduction

**Figure 1:**
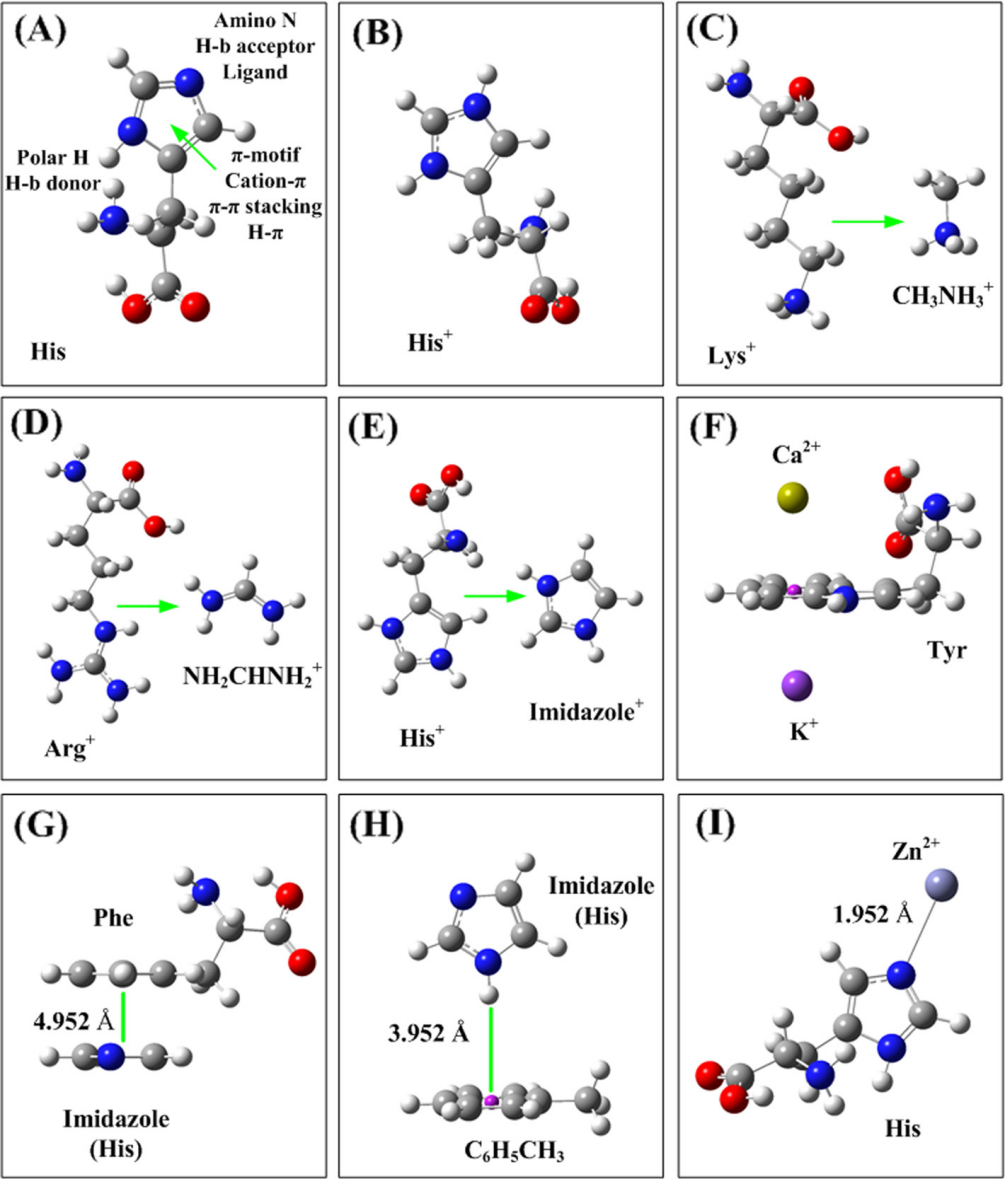
**The optimized geometries of amino acids and the structures of four interaction types.** (**A**) The optimized geometry and the interaction contributors of histidine (His). (**B**) The optimized geometry of protonated histidine (His^+^). The protonated imidazole is an organic cation in the cation-π interactions with other aromatic amino acids. (**C**) The protonated Lys is simplified as CH_3_NH_3_^+^. (**D**) The protonized Arg^+^ is simplified as CHNH_2_NH_2_^+^. (**E**) The protonated His^+^ is simplified as the protonated imidazole C_3_N_2_H_5_^+^. (**F**) The interaction structure of cation-π interaction. The cation could be at the upside or downside of the aromatic plane. (**G**) The interaction structure of π-π stacking interaction between Phe and His (simplified as the imidazole). In the π-π stacking interaction the two aromatic planes are stacking in parallel. (**H**) The hydrogen-π interaction structure between His (imidazole) and aromatic motif. The polar hydrogen atom of His perpendicularly points to the π-plane. (**I**) The coordinate bonding interaction structure between His and metallic cation.

The 20 natural amino acids are the building blocks of three dimensional protein structures. Each of them has its unique structural characters and physicochemical properties, and plays irreplaceable role in biochemistry and biological functions of proteins. Among the 20 natural amino acids histidine (His, H) may be the most versatile actor in the protein architectures and bioactivities [1-4]. The versatility of histidine in molecular interactions arises from its unique molecular structure [5]. The side chain imidazole of histidine is an aromatic motif; an ionizable group with the acidic ionization constant around pK_a_=6.5; a coordinating ligand of metallic cations (for example, Ca^2+^ and Zn^2+^); and a hydrogen bond donor and acceptor. The unique structure of histidine makes it plays multiple roles in the molecular interactions. The roles of histidine in molecular interactions are even complicated by pH condition and its two protonation forms, the neutral form and the protonated form [6]. Figure 1 shows the optimized structures of histidine in the neutral form (**A**) and in the protonated form (**B**). The functional groups of histidine and their interaction functions are illustrated in Figure 1.

The molecular interactions of histidine with other amino acids and metallic cations in proteins can be classified into the following five types. (1) *Cation*-*π interaction*[7-9]. The side chain imidazole of His is an aromatic ring. Histidine can take part in the cation-π interactions as the aromatic motif with metallic cations or organic cations (protonated amino acids, Lys^+^ and Arg^+^) [7,9-11]. On the other hand, the protonated His^+^ is an organic cation, which can join the cation-π interactions as an organic cation with other aromatic amino acids (Phe, Tyr, and Trp) [12-16]. (2) *π*-*π stacking interaction*[17-20]. The imidazole structure of histidine side chain is a conjugative π-plane, which can make π-π stacking interactions with the aromatic side chains of other amino acids (Phe, Tyr, and Trp) [20,21]. (3) *hydrogen*-*π interaction*[22,23]. The polar hydrogen atom of histidine can form hydrogen-π bond with other aromatic amino acids in ‘T’ orientation. (4) *Coordinate bond interaction*[3,24,25]. The basic nitrogen atom in the imidazole of histidine has a lone electron pair that make it a coordinate ligand of metallic cations, such as Zn^2+^ and Ca^2+^[26,27]. (5) *Hydrogen bond interaction*[28-31]. The polar hydrogen atom of the imidazole is a hydrogen-bond donor, and the basic nitrogen atom is a hydrogen-bond acceptor.

In protein interactions the roles of histidine are complicated by the five interaction types and two protonation forms. The unique behaviors of histidine have been discussed in literatures from different aspects [7,32]. However, the quantitative interaction energies of five interaction types and the factors affecting the interaction energies still need more investigations. The influences of five interaction types to the protonation form (and pK_a_ value) of histidine are still unclear. In this study the multiple roles of histidine in molecular interactions are quantitatively studied using quantum chemical calculations, and the factors, which influence the interaction energies and pK_a_ value of histidine in proteins, are analyzed in detail.

## Methods and materials

Quantum chemistry is a powerful tool in chemical and biochemical studies [33,34]. The cation-π and π-π stacking interactions have been studied by many authors using several QM methods [35,36]. The DFT method B3LYP is a widely used method in organic chemistry and biochemistry because of its higher accuracy and less computational workload [37,38]. However, in past twenty years the DFT methods were found to fail in describing the molecular dispersion interactions [39-42]. On the other hand, the more advanced coupled-cluster with single and double CCSD and triple excitations CCSD(T) methods [43-46] are able to evaluate the dispersion interaction well. However, such sophisticated methods take much more computer cpu time than that of the DFT methods. A computational comparison of the three methods (B3LYP, CCSD, and CCSD(T)) to the five interaction types (mentioned in the introduction section) is performed. The results of the comparison calculations are listed in Table 1.

**Table 1:** **Comparison of three methods** (**DFT**, **CCSD**, **and CCSD**(**T**)) **for five interaction types** (**cation**-**π**; **π**-**π staking**; **hydrogen**-**π**; **hydrogen bond**; **and metallic cation**-**coordinate interaction**

**Interaction pair**	**Molecule**	**B3LYP/6-31+G(d,p)**		**CCSD/6-31+G(d,p)**	
		**E**_**int**_**(kcal/mol)**	**R(Å)**	**E**_**int**_**-kcal/mol)**	**R(Å)**
^a^ π-π stack	C_6_H_6_-C_6_H_6_	+0.100	7.874	−1.883	4.262
^b^ H-π	C_6_H_5_CH_6_-Imid	−2.444	3.616	−5.897	3.324
^c^ H-b	NMA-NMA	−5.827	2.186	−6.023	2.022
Coordinate	Imid-Na^+^	−38.045	2.267	−36.788	2.317
^d^ Cation-π	C_6_H_6_CH_3_-H_3_O^+^	E_int_(kcal/mol)	R(Å)	CPU time	
B3LYP/6-31+G(d,p)		−17.791	2.781	1.08 hours	
CCSD/6-31+G(d,p)		−18.147	2.781	50 days	
CCSD(T) /6-31+G(d,p)		−18.872	2.781	86 days	

^a^ DFT method B3LYP/6-31+G(d,p) cannot yield attractive interaction energy for C_6_H_6_-C_6_H_6_ π-π stacking interaction, failing in describing the dispersion dominated π-π stacking interactions.

^b^ ‘H-π’ indicates the interaction between polar hydrogen atom with aromatic molecule in ‘T’ orientation, in which the dispersion energy contributes more than 50%. The energy difference between CCSD and DFT calculations is defined as the dispersion contribution.

^c^ ‘H-b’ indicates the common hydrogen bond interaction, which is the MO-coordinated and charge dominated interaction.

^d^ In the cation-π interactions the electrostatic interactions and MO orbital coordinate interactions make the main contributions, and the dispersion contribution is less than 10%.

From the data in Table 1 we find that the DFT method B3LYP cannot yield attractive interaction energy in C_6_H_6_-C_6_H_6_ π-π stacking interaction, completely failing in describing the π-π stacking interactions, which are dispersion dominated phenomenon. On the other hand the higher level method CCSD calculation produces attractive C_6_H_6_-C_6_H_6_ π-π stacking energy −1.883 kcal/mol. In this study the energy differences between B3LYP and CCSD are used as the dispersion contribution in the molecular interaction energies. In the hydrogen-π interaction more than 50% interaction energy is from the dispersion contribution. The interaction energies of other three interaction types (cation-π interactions, common hydrogen bond interactions, and metal cation-His coordinate interactions), obtained by using B3LYP and CCSD methods, have no remarkable difference. In above three interaction types the electrostatic (charge) interactions and orbital coordinate interactions make the main contributions, and the contribution of dispersion interactions are less than 10% [8,41,42]. In the C_6_H_6_CH_3_-H_3_O^+^ cation-π calculations the CPU time of three methods (B3LYP, CCSD, and CCSD(T)) are 1.08 hours, 50 days, and 86 days, respectively. However, the energy difference of cation-π interaction between B3LYP and CCSD(T) is only 1.08 kcal/mol, less than 6%.

In this study the π-π stacking interactions and the hydrogen-π interactions are calculated using CCSD/6-31+G(d,p) method, and the B3LYP/6-31+G(d,p) is used in the calculations of cation-π interactions and ligand-cation coordinate interactions. In recent years great efforts are made to make up the shortcoming of DFT in dispersion interactions, including design of new functional [47], or empirical correction terms [41,42,48-50]. , In this study the missing dispersion energies in DFT calculations are corrected by an empirical method suggested by Du et al [42]. The interaction energies in solutions are calculated by using the polarizable continuum model (PCM) [50-53].

In this study most molecule monomers are optimized by using CCSD/6-31+G(d,p) methods. Some large amino acids, such as Tyr and Trp, first are optimized at B3LYP/6-31+G(d,p) level, then the side chains are optimized at CCSD/6-31+G(d,p) level. The geometry parameters of side-chain, obtained from CCSD calculations, are combined with the parameters of DFT optimizations. In this study the protonated His^+^ is simplified as the protonated imidazole (C_3_N_2_H_4_^+^), protonated Arg^+^ is simplified as CHNH_2_NH_2_^+^, and the protonated amino acid Lys^+^ is simplified as CH_3_NH_3_^+^, respectively, as shown in Figure 1C, D, and E. The structures of four interaction types (cation-π interaction, π-π stacking interaction, hydrogen-π interaction, and coordinate bond interaction) of His are shown in Figure 1F, G, H, and I, respectively. Usually amino acids have several stable structural conformations with different energies. In proteins the orientations of residue side chains and the structural conformations of peptide backbone are innumerous. The optimized structures of amino acids, shown in Figure 1, are only one of the possible conformations. In Figure 1 F the metallic cation can be put at the upside or at the downside of the aromatic planes. In the ‘Upside’ structure the cation-π interaction may be complicated by the interaction elements in peptide backbone. On the other hand, the ‘Downside’ structure is less affected by other interaction elements. In this study we focus on the ‘pure’ cation-π interactions, the ‘Downside’ structures. All calculations are performed on Sugon-5000A computer using Gaussian 09 software package [54]. The detailed geometrical parameters of optimized molecular structures are stored in supporting material (Optimized-Mol.zip).

## Results

In this section all calculation results are reported and summarized using tables and figures. Brief comparisons and illustrations are provided following the calculation results. Four interaction types (cation-π, π-π stacking, hydrogen-π, and coordinate bond interaction) of histidine with other amino acids and metallic cations are calculated in gas phase and in solutions (water, acetonitrile, and cyclohexane). The hydrogen bonding interaction of histidine is not included in this study, because it is a familiar and well studied interaction type.

### Cation-π interactions of Histidine

The cation-π interaction energies of His are summarized in Table 2. In the up part of Table 2 the His is the aromatic motif in neutral form. The cation-π interaction energies are different when the cations are at the downside and upside of the aromatic π-plane, because the interaction environments are different at the two sides (see Figure 1F). The cation-π energy (−147.4 kcal/mol) of Zn^2+^ is much larger than other cations, because the 3d valence orbitals of Zn^2+^ can make stronger bonding MO with the imidazole π-MO of histidine. In gas phase the cation-π interaction energies of His with organic cations (protonated amino acids Lys^+^ and Arg^+^) are in the range −8 to −9 kcal/mol, stronger than the common hydrogen bonds of water (−5 to −6 kcal/mol) [55,56]. However, the cation-π interaction energies of His are smaller than that of other three aromatic amino acids (Phe, Tyr, and Trp) because of the smaller π-system size [57,58]. In the lower part of Table 2 the protonated histidine (His^+^) is the cation in the cation-π interactions. The cation-π interaction energy of His^+^-Trp is −13.6 kcal/mol, larger than other two interaction pairs (His^+^-Tyr and His^+^-Phe), because of the larger aromatic size of Trp.

**Table 2:** **Cation**-**π interaction energies between amino acid His and cations in gas phase**

**His (Aromatic motif)**	**Downside**		**Upside**	
	**Energy **^**a**^	**Length **^**b**^	**Energy **^**a**^	**Length **^**b**^
Na^+^	−16.457	2.461	−10.478	2.420
K^+^	−10.066	2.957	−2.358	2.925
Ca^++^	−54.331	2.493	−45.771	2.483
Zn^2+^	−147.406	2.137	−144.355	2.820
Lys(CH_3_NH_3_^+^)	−8.193	3.107 ^c^	−0.198	3.069 ^c^
Arg(CHN_2_NH_2_^+^)	−9.268	3.911 ^c^	−2.918	3.883 ^c^
His^+^	Downside		Upside	
(Organic cation)	Energy ^a^	Length ^d^	Energy ^a^	Length ^d^
Phe	−7.809	3.269	−3.613	4.809
Tyr	−7.887	3.256	−3.655	4.799
Trp	−13.642	3.166	−12.057	3.276

^a^ Energies are in kcal/mol, calculated using B3LYP/6-31+G(d,p) method.

^b^ Angstrom (Å).

^c^ Distance from N of CH_3_NH_3_^+^ (or CHNH_2_NH_2_^+^) to the center of imidazole ring.

^d^ Distance from N of imidazole to the aromatic center of amino acids (Phe, Tyr, and Trp).

The cation-π interaction energies in three solvents (water, acetonitrile, and cyclohexane) are listed in Table 3. The cation-π interaction energies decrease sharply with the increase of solvent dielectric constants ε. Generally speaking, in gas phase cation-π interaction energies of metallic cations are larger than that of organic cations (protonated amino acids), however, in the aqueous solution, the cation-π interaction energies of organic cations are larger than that of metallic cations.

**Table 3:** **Cation**-**π interaction energies between histidine** (**His**) **and cations in three solvents** (**water**, **acetonitrile**, **and cyclohexane**)

	**Water (ε**=**78**.**39)**		**Acetonitrile (ε**=**35**.**9)**		**Cyclohexane (ε**=**2**.**0)**	
**Cation**–**His**	**Energy **^**a**^	**Length **^**b**^	**Energy**	**Length**	**Energy**	**Length**
Na^+^	−0.262	3.556	−0.182	3.536	−3.145	2.777
K^+^	−0.019	4.007	−0.160	3.996	−5.433	3.120
Ca^++^	+0.087	3.835	−0.568	3.797	−8.898	2.582
Zn^2+^	+0.009	3.835	−0.828	2.885	−41.442	2.115
Lys(CH_3_NH_3_^+^)	−0.391	3.961	−0.324	3.944	−2.310	3.245
Arg(CHN_2_NH_2_^+^)	−0.858	4.148	−0.842	4.133	−3.938	3.979
His^+^–π						
Phe	−0.602	2.499	−0.965	2.493	−3.429	2.305
Tyr	−0.884	2.511	−0.924	2.505	−3.421	2.299
Trp	−1.368	2.412	−1.512	2.401	−6.679	2.203

^a^ Energies are in kcal/mol, calculated using B3LYP/6-31+G(d, p) + PCM method.

^b^ Angstrom (Å).

**Figure 2:**
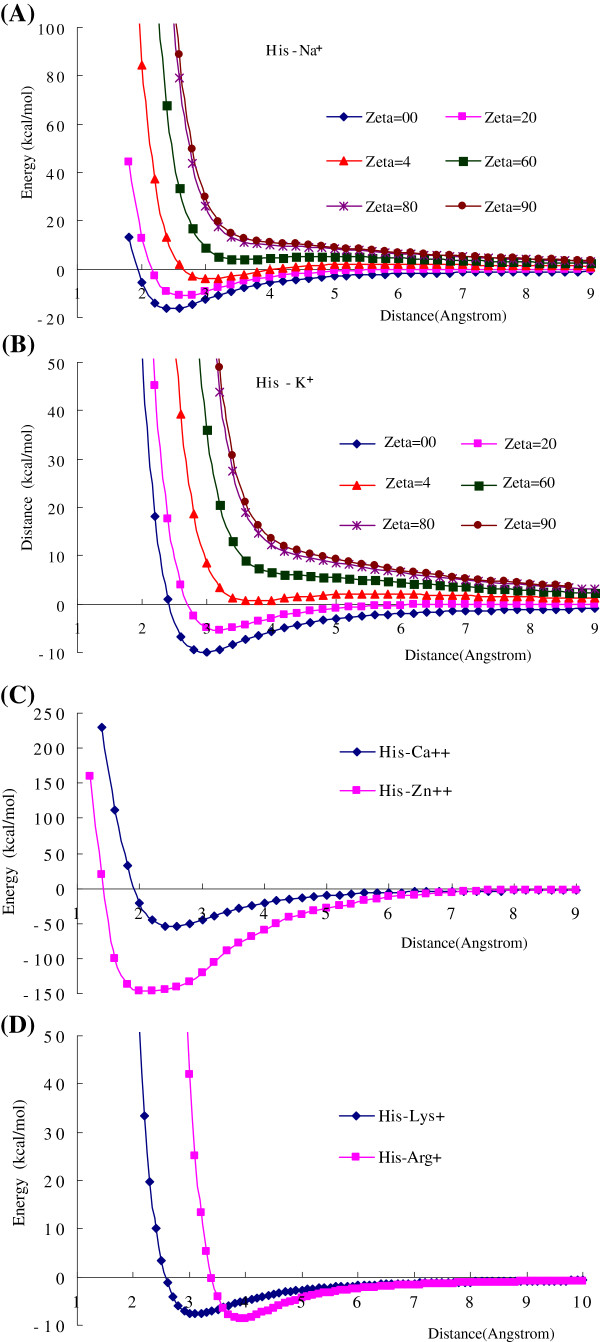
**The cation**-**π interaction energies of histidine** (**His**) **with metallic cations and organic cations.** (**A**) The cation-π interaction energies of His–Na^+^ as the function of distance R and orientation angle θ. (**B**) The cation-π interaction energies of His–K^+^ as the function of distance R and orientation angle θ. The cation-π interactions are distance and orientation dependent. The most favorable direction is perpendicular to the center of π-plane. (**C**) The cation-π interaction energies of His–Ca^2+^ and His–Zn^2+^ as the function of distance between cation and the aromatic center of His. (**D**) The cation-π interaction energies of His–Lys^2+^ and His–Arg^+^ as the function of distance between cation and the aromatic center of His. All calculations are performed by using B3LYP/6-31+G(d,p) method.

The cation-π interaction energies are distance and orientation dependent. Figure 2 shows the cation-π energy curves of several cation-π interaction pairs as the functions of distance (R) and orientation angle (θ). Figure 2A and B are the interaction energies of His-Na^+^ and His-K^+^ as the functions of distance (from the cation to the center of imidazole ring) and the orientation angle (between the interaction direction and the perpendicular direction). The strongest interaction is at the perpendicular direction (θ=0°) to the π-plane. Figure 2C and D show the interaction energy curves of His-Ca^2+^, His-Zn^2+^, His-Lys^+^ and His-Arg^+^ at the perpendicular direction. The curve shapes of cation-π interaction energies are similar to the common van der Waals interactions, however, the attractive interaction regions are broader, and at the short distance the repulsive interactions are softer than the common van der Waals interactions.

**Figure 3:**
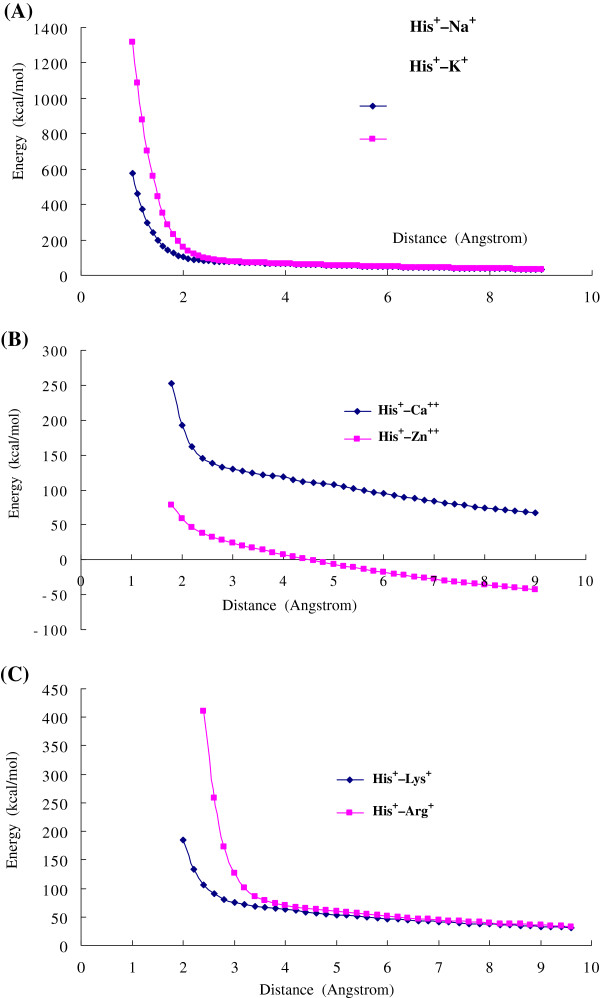
**The repulsive cation**-**π interactions between protonated histidine** (**His**^+^) **and cations.** (**A**) The repulsive cation-π interactions between protonated histidine (His^+^) and cation Na^+^ and K^+^. At short distance the repulsive interaction energies are very strong, then the energies decrease with the distance R. (**B**) The repulsive cation-π interactions between protonated histidine (His^+^) and cation Ca^2+^ and Zn^2+^. At short distance the curves are softer than that of Na^+^ and K^+^. At long distance (>5Å) the interaction of His^+^–Zn^2+^ turns to attractive, which may arise from the long interaction range of 3d valence orbitals of Zn^2+^. (**C**) The repulsive cation-π interactions between protonated histidine (His^+^) and organic cations Lys^+^ and Arg^+^. All calculations are performed by using B3LYP/6-31+G(d,p) method.

After the His is protonated (His^+^) there are no attractive cation-π interactions between His^+^ and the metallic cations (e.g., Na^+^, K^+^, and Ca^2+^) and the organic cations (Arg^+^ and Lys^+^), instead of repulsive interactions. Figure 3 shows the repulsive cation-π interactions between the protonated histidine (His^+^) and cations as the function of distance R. In protein structures the histidine frequently and reversibly transforms from neutral form to protonated form [6,59,60]. Based on our calculations the proton transformation in His may switch the cation-π interactions from attractive to repulsive. Actually the His^+^–cation interactions are the combination of repulsive electrostatic interaction between two cations and the attractive cation-π interaction between cation and aromatic motif. In Figure 3A in short distance the repulsive interactions of His^+^ with Na^+^ and K^+^ are steep, because the strong repulsive electrostatic forces exceed the attractive cation-π forces. However, the repulsive interactions of His^+^ with Ca^2+^ and Zn^2+^ are softer at the short distance, because the attractive cation-π forces of Ca^2+^ and Zn^2+^ are larger than that of Na^+^ and K^+^. Particularly at longer distance the interaction of His^+^–Zn^2+^ turns to attractive, which may arise from the long interaction range of 3d valence orbitals of Zn^2+^ in cation-π interactions.

### π-π stacking interactions of histidine

The π-π stack is referred to the face to face interactions between two or more aromatic molecules. In the 20 natural amino acids 4 of them contain aromatic side chains (Phe, Tyr, Trp, and His). The π-π stacking interactions in proteins are a controversial research topic [17-21]. Our and other authors’ comparison calculations revealed that π-π stacking interactions are dispersion-dominated phenomenon [43-46]. For the neutral (uncharged and non polar) π-π stacking interactions the B3LYP method cannot yields attractive energies, and DFT fails in describing dispersion energies. Our calculation results using CCSD/6-31+G(d,p) are summarized in Table 4. In proteins the strength of the π-π stacking interactions between neutral His and other aromatic amino acids (Phe, Tyr, and Trp) are in the range from −3.0 to −4.0 kcal/mol, higher than the C_6_H_6_-C_6_H_6_ π-π stacking energy (−1.88 kcal/mol) [57], because the π-π interaction energies between aromatic amino acids may contain the contributions of hydrogen-π interactions [61,62], which will be discussed in next section. The π-π stacking interaction energies between the protonated histidine (His^+^) and other aromatic amino acids are in the range from −3.6 to −8.4 kcal/mol, remarkably larger than that of neutral His.

**Table 4:** **The π**-**π stacking interaction energies between His and aromatic amino acids in gas phase**

	**B3LYP**		**CCSD**	
^**a **^**His**	**Energy **^**b**^	**Length **^**c**^	**Energy **^**b**^	**Length **^**c**^
Imid-Phe	−0.093	5.754	−3.084	3.822
Imid-Tyr	−0.098	5.331	−3.463	3.714
Imid-Trp	−0.535	4.938	−4.035	3.740
^d^ His-Arg^+^	−2.402	3.914	−5.043	3.522
^a^ His^+^				
Imid^+^-Phe	−1.696	4.224	−3.683	3.633
Imid^+^-Tyr	−1.733	4.082	−4.143	3.564
Imid^+^-Trp	−6.514	3.798	−8.425	3.478

^a^ Histidine (His) is simplified as the aromatic motif imidazole (Imid).

^b^ Energies are in kcal/mol, calculated by using CCSD/6-31+G(d,p) and  B3LYP/6-31+G(d,p) methods.

^c^ The distance between two π-planes, in angstrom (Ǻ ).

^d^ The motif CHNH_2_NH_2_^+^ of protonated Arg^+^ forms a π-plane.

The π-π stacking energies increase with the size of π-system. In Table 4 the π-π stacking energy (−4.035 kcal/mol) of His-Trp is larger than that of His-Phe and His-Tyr because of the larger π-system of Trp. In DNA the π-π stacking interactions have larger contributions than in proteins [41,47-49]. The protonated amino group (CHNH_2_NH_2_^+^) of Arg^+^ forms a π-plane, and the larger π-π stacking energy (−5.0432 kcal/mol) of His-Arg^+^ may partially from the cation-π interaction. The lower part of Table 4 lists the π-π stacking interaction energies between the protonated His^+^ and three aromatic amino acids (Phe, Tyr, and Trp), which are remarkably larger than that in the up part of Table 4.

### Hydrogen-π interactions of histidine

The hydrogen-π interaction is the interaction between polar hydrogen atom and π-electron density of aromatic molecule [61,62]. In contract to the hydrogen-π interaction, the common hydrogen bond is the interaction between polar hydrogen and electron density of electronegative elements (such as oxygen and nitrogen). Unlike π-π stacking interactions, in which the π-planes of two aromatic amino acids are in parallel orientation, the hydrogen-π interactions of histidine and other aromatic amino acids take ‘T’ orientation. In proteins when the polar hydrogen atom of histidine perpendicularly points to the aromatic ring of other amino acids, the hydrogen-π interaction happens. The hydrogen-π interaction energies of His with other aromatic amino acids (Phe, Tyr, and Trp) are listed in Table 5.

**Table 5:** **The hydrogen**-**π interaction energies between His and aromatic amino acids in gas phase**

	**B3LYP/6-31+G(d,p)**		**CCSD/6-31+G(d,p)**	
^**a **^**His**	**Energy **^**b**^	**Length **^**c**^	**Energy **^**b**^	**Length **^**c**^
Imid-Phe	−2.735	2.594	−5.663	2.594
Imid-Tyr	−2.599	2.578	−5.637	2.578
Imid-Trp	−3.679	2.548	−7.907	2.548

^a^ Histidine (His) is simplified as the aromatic motif imidazole (Imid).

^b^ Energies are in kcal/mol, calculated by using CCSD/6-31+G(d,p) method.

^c^ The distance between two π-planes, in angstrom (Ǻ ).

Comparing the data in Table 4 and Table 5, the energies of hydrogen-π interactions are larger than the corresponding energies of π-π stacking interactions. In proteins the energies of hydrogen-π interactions are in the range −5 to −8 kcal/mol, comparable to the common hydrogen bond interactions (−4 to −6 kcal/mol). Actually, the π-π stacking interaction energies of aromatic amino acids contain the contributions of hydrogen-π interactions from the polar hydrogen atoms in His and in Tyr.

### Coordinate bonding interactions between His and cations

**Figure 4:**
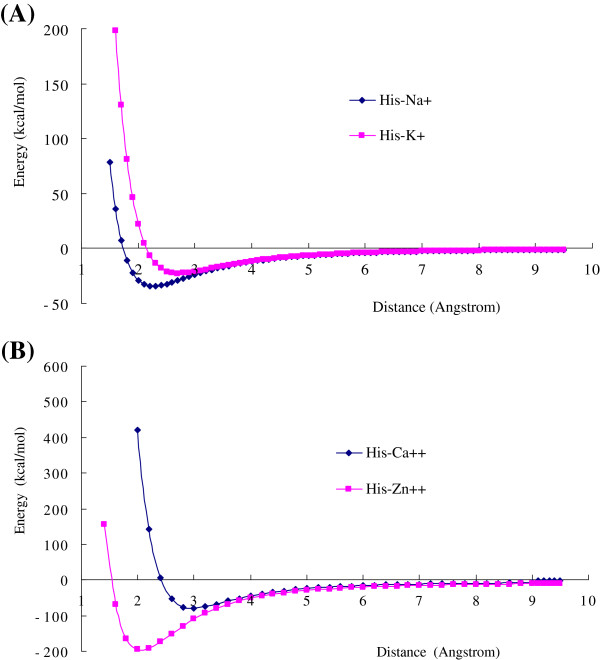
**The coordinate interaction energies of His with metallic cations as the function of distance R.** (**A**) The coordinate bonding interaction curves of His–Na^+^ and His–K^+^. (**B**) The coordinate bonding interaction curves of His–Ca^2+^ and His–Zn^2+^. The interaction energies of coordinate bonding interactions are larger than other three interaction types (cation-π interaction, hydrogen-π interaction, and π-π stacking interaction). The coordinate interaction of His–Zn^2+^ is a long range interaction, and the energy is as high as −195 kcal/mol. All results are calculated at B3LYP/6-31+G(d,p) level.

The basic nitrogen atom in imidazole ring of His bears a lone electron pair, which may form coordinate bond with metallic cations, such as Ca^2+^, Zn^2+^, and Cu^2+^. The coordinate interaction of His is a unique molecular interaction type in the 20 natural amino acids. The coordinate interaction energies of His with some metallic cations (Na^+^, K^+^, Ca^2+^, and Zn^2+^) are listed in Table 6. The energy curves of His-cation coordinate interactions as the function of distance are shown in Figure 4. Comparing the Table 6 with Table 2, 4 and 5, the His-cation coordinate interaction energies are much stronger than the cation-π interactions and the π-π stacking interactions. The interaction energy (−195.2164 kcal/mol) of His-Zn^2+^ is higher than all other interaction pairs, because the 3d valence orbitals of Zn^2+^ can make stronger bonding MO with the lone electron pair of nitrogen atom in imidazole. The metallic cations often play important role in the catalytic activity of enzymes in biology. Therefore the coordinate interactions of His with metallic cations are significantly important in protein science and biochemistry.

**Table 6:** The coordinate bonding interaction energies between His and metallic ations in gas phase and in solutions

**His**	**Gas phase**		**Water (ε** =**78**.**39)**		**Acetonitrile (35.9)**		**Cyclohexane (2.0)**	
(neutral)	Energy ^a^	Length ^b^	Energy	Length	Energy	Length	Energy	Length
His–Na^+^	−34.402	2.272	−5.929	2.386	−6.330	2.383	−18.595	2.325
His–K^+^	−22.807	2.722	−3.917	2.836	−4.177	2.836	−12.525	2.781
His–Ca^2+^	−80.000	2.367	−8.580	2.557	−9.185	2.554	−36.307	2.442
His–Zn^2+^	−195.216	1.952	−16.842	1.952	−33.627	1.950	−92.137	1.904

^a^ Energies are in kcal/mol, calculated by using B3LYP/6-31+G(d,p) +PCM method.

^b^ Angstrom (Ǻ ).

**Figure 5:**
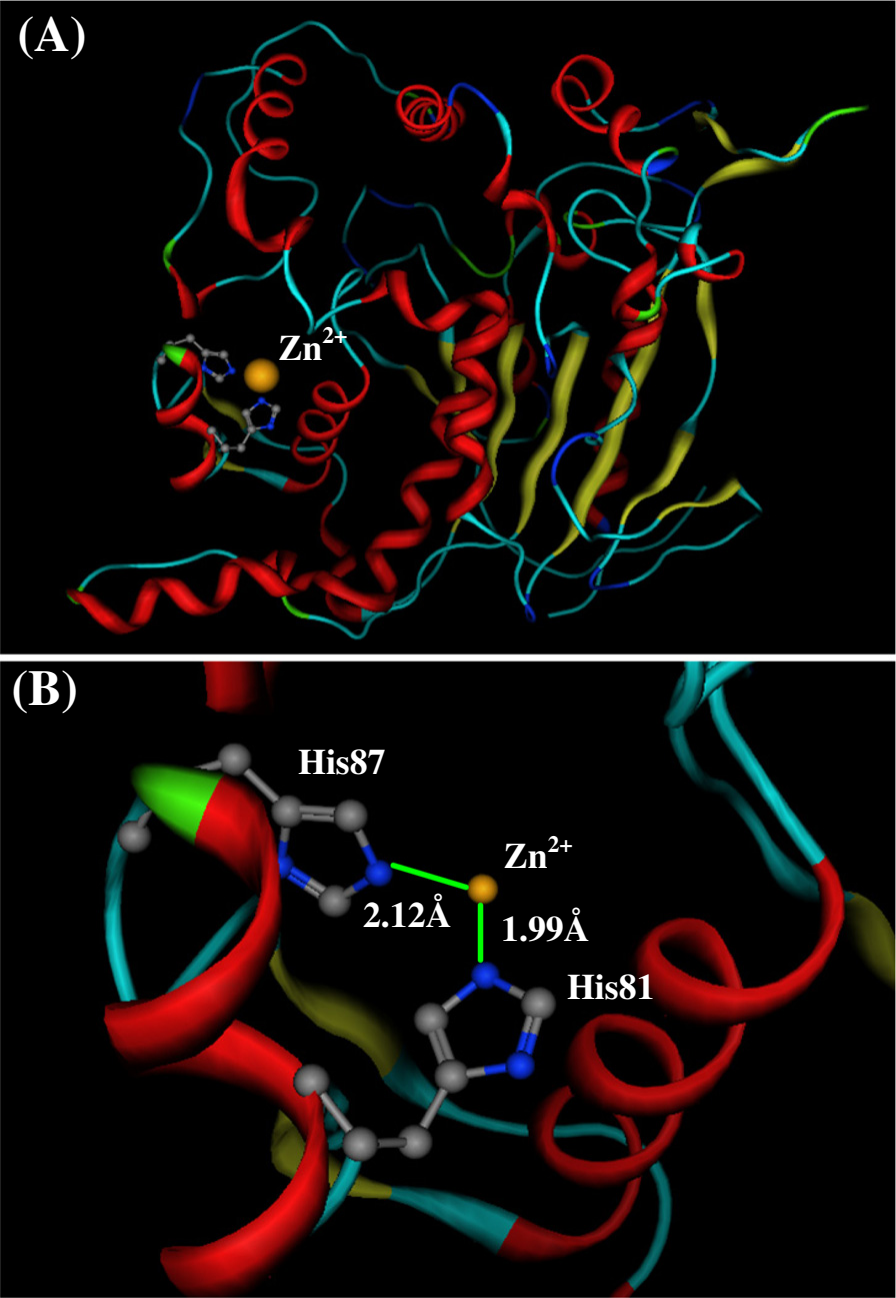
**The coordinate bonding interaction between His and Zn**^**2 **+^**in T1 lipase (PDB code: 1JI3).** (**A**) The location of His81, His87, and Zn^2+^ in the T1 lipase structure. (**B**) The coordinated bonds between His81 and Zn^2+^, and between His87 and Zn^2+^. The coordinate bond lengths of His81–Zn^2+^ and His87–Zn^2+^ are 2.12 Å and 1.99 Å, respectively, very close to the optimized distance (1.9519 Å).

Although alkali metallic cations (Na^+^ and K^+^) are the most abundant elements in living systems, the transition metallic cations (e.g., Zn^2+^, Cu^2+^, and Fe^2+^) often play important role in the bioactivities of proteins and enzymes [3,63]. In many cases the transition metallic cations bind at the host proteins through the coordinate bonds. For example, the extracellular lipase (T1 lipase) from Geobacillus zalihae strain T1 is a thermoalkalophilic enzyme [13,64]. In the crystal structure of T1 lipase (PDB code 1JI3) a zinc cation Zn^2+^ binds with two histidines (His81 and His87) in the host pocket, as shown in Figure 5, which is one of the most conservative motif in the lipase family. The interaction structure between Zn^2+^ and two His is in the best orientation and distances for the coordinate interaction. The coordinate bond lengths of Zn^2+^ with His81 and His87 are 2.12 Å and 1.99 Å, respectively, very close to the optimized distance (1.9519 Å) in Table 6.

## Discussion

Histidine is an ionizable amino acid with the acidic ionization constant around pK_a_=6.5, very close to neutral. An interesting finding in this study is that the protonation of histidine has closely relationship with the interaction types. The cation-π interactions of neutral histidine (His) are attractive, and the cation-π interactions of protonated histidine (His^+^) are repulsive. A reasonable deduction is that pH condition can reversibly switch the cation-π interactions of histidine from attractive to repulsive. Vice versa, the cation-π interactions can affect the two protonation forms of histidine. In proteins the pK_a_ value of His can change in a broad range due to the influence of interaction environment, and histidine can play the roles of both proton donor or acceptor [58,65,66]. The stronger attractive cation-π interaction can make the pK_a_ value of His lower, and the lower pH condition may turn the cation-π interaction from attractive to repulsive. For the same reason, other interaction types (coordinate interaction, hydrogen-π interaction, hydrogen bond and the π-π stacking interaction) may also affect the pK_a_ value of histidine to some degree [60].

In protein hydrolysis reactions the pK_a_ value of His is a critically important property. In the catalytic triads of lipase, the basic nitrogen of histidine is used to abstract a proton from threonine, serine, or cysteine to activate it as a nucleophile. In carbonic anhydrases, a histidine proton shuttle is utilized to rapidly transport protons away from a zinc-bound water molecule to quickly regenerate the active form of the enzyme [67,68]. In the histidine proton shuttle, histidine abstracts a proton with its basic nitrogen to make a positively-charged intermediate, and then use another molecule, a buffer, to extract the proton from its acidic nitrogen. Our study illustrates that in the proton shuttle procedure, the histidine is not working by itself alone, but with the collaboration of environmental residues through the multiple interactions that affect the pK_a_ value of histidine.

## Conclusion

Based on our calculation results the energy order of five interaction types (cation-π interaction, π-π stacking interaction, hydrogen-π interaction, hydrogen-bond interaction, and coordinate bond interaction) is as follows, E_coor_>E_cation-π_>E_H-π_≈E_H-b_>E_π-π_. The coordinate interaction (E_coor_) of His with metallic cations is the strongest interaction with long interaction distance, followed by the cation-π interaction (E_cation-π_). In the cation-π interactions, when His is in neutral form (unprotonated), interaction energy is attractive. However, when His is protonated, the interaction energy turns to repulsive. The π-π stacking interactions are the π-plane to π-plane interactions, with much more interaction conformations than other interaction types. In proteins the energies of π-π stacking interactions (E_π-π_) can change in a broad range, because of different interaction orientations. The π-π stacking interactions between neutral His and aromatic amino acids (Phe, Tyr, and Trp) are in the range −3.0 to −4.0 kcal/mol, significantly larger than the van der Waals interactions. However, the π-π stacking energies of protonated histidine (His^+^) are much larger than the energies of neutral His.

The interaction strength of cation-π interactions in solutions is a controversial research topic [17,65,69]. Based on our calculations by using PCM method, the energies of cation-π interactions decrease sharply with the increase of the dielectric constant ε of solvents. In gas phase the cation-π interaction energies of metallic cations are larger than that of organic cations (Lys^+^ and Arg^+^). However, in solutions of polar solvents (water and acetonitrile) the cation-π interaction energies of organic cations (protonated amino acids) are lager than that of metallic cations. The PCM is a continuum medium model [50-53]. The calculated values of PCM may be not very accurate, but the qualitative order is meaningful. In aqueous solution the cation-π interactions between protonated amino acids and aromatic amino acids may be more important than that of metallic cations [17,69,70]. However, this does not mean that the cation-π interactions of metallic cations are not important in solutions. In aqueous solution the hydrophilic residues are explored on the surface, and the hydrophobic residues are hidden in the core region of protein structures. In the hydrophobic pockets of proteins the dielectric constants are smaller than that in bulk solution. Therefore, the cation-π interactions are still working in the hydrophobic pockets and in core region of proteins.

## Competing interests

We declare that there are no any competing interests.

## Authors’ contributions

All authors contributed equality for the development of the manuscript. RBH and QSD designed the research scheme and wrote the article. SML did most calculations, JZM and ZWP performed the data collection and analysis. All authors read and approved the final manuscript.
